# Molecular characterization of tetracycline and vancomycin-resistant *Enterococcus faecium* isolates from healthy dogs in Egypt: a public health threat

**DOI:** 10.1186/s12864-023-09708-4

**Published:** 2023-10-12

**Authors:** Khaled A. Abd El-Razik, Eman S. Ibrahim, Amany A. Arafa, Riham H. Hedia, Abdelgayed M. Younes, Mahmoud H. Hasanain

**Affiliations:** 1 Department of Animal Reproduction, Veterinary Research Institute, National Research Centre, Dokki, Giza, 12622 Egypt; 2 Department of Microbiology and Immunology, Veterinary Research Institute, National Research Centre, Dokki, Giza, 12622 Egypt; 3 Department of Hydrobiology, Veterinary Research Institute, National Research Centre, Dokki, Giza, 12622 Egypt

**Keywords:** *Enterococcus faecium*, Dogs, Tetracycline, Vancomycin, Bacteriocins, One health

## Abstract

**Background:**

Vancomycin-resistant *enterococci* (VRE) are among the most common causative pathogens for nosocomial infections worldwide. Moreover, strains of VRE have been isolated from several domestic livestock in Egypt.

**Methods:**

This study examined if healthy dogs are a potential source of VRE infection by isolating and characterizing *Enterococcus faecium* strains from stool samples on a morphological basis and biochemical activities. Subsequently, it was confirmed by genotypic characterization using polymerase chain reaction (PCR), followed by the detection of antibiotic resistance genes, virulence determinants, and genes contributing to enterocin production by PCR. Furthermore, the phylogenetic relationships among *vanB* and *tetL* genes were analyzed.

**Results:**

All ten fecal samples were identified as *E. faecium* and confirmed by PCR. In addition, 90% of the isolates tested were positive for the virulence genes *gelE* and *esp*, and all the isolates tested were positive for the antibiotic resistance genes *tetL* and *vanB*. Only three of the five enterocin genes examined were detected. *Ent As-48*, *bacteriocin 31*, and *Ent L50* were identified in 100%, 80%, and 60% of the samples, respectively.

**Conclusion:**

Dogs should be regarded as a reservoir of *E. faecium* that carries vancomycin resistance and virulence determinants that may affect public health in Egypt, considering a "One Health" task force approach to restrict their spread.

## Introduction

An ancient relationship between humans and dogs evolved from a working animal to welfare use as a companion animal. Although they can improve their owners' physical and mental health, they can spread disease. The first study in dogs reported the existence of multidrug-resistance Shiga-toxigenic *Escherichia coli* in Egypt [1]. The One Health effort is a global approach to partnerships in all facets of health care for people, animals, plants, and the environment, providing a basis for combating major public health threats [2]. *Enterococci* are commensals of the gastrointestinal tract of both humans and animals and are a significant cause of nosocomial infections [3]. Companion animals (such as dogs and cats) may act as asymptomatic reservoirs for virulent and multidrug-resistant (MDR) enterococcal species (*E. faecalis* and *E. faecium*) [4]. A high percentage of MDR *E. faecalis* strains isolated from companion animals have been described [5]. However, intestinal carriage of vancomycin-resistant *enterococci* (VRE) was rare in healthy dogs [6]. Many studies have focused on detecting VRE in swine, poultry, and food-producing animals, but the epidemiology of *E. faecalis* and *E. faecium* in dogs is not well described [7].

Enterococcal strains can produce bacteriocins, and several of these probiotic proteins have been purified and genetically characterized to control pathogenic bacteria [8]. Despite their benefits, certain enterococcal strains are associated with their pathogenic characteristic as opportunistic bacteria. *Enterococci* possess several virulence genes, including *asa1* (encoding aggregating substance), *gelE* (encoding gelatinase), *esp* (encoding enterococcal surface protein), and *ace* (encoding adhesion of collagen). Enterococcal surface proteins (*esp*), hyaluronidase (*hyl*), and collagen-binding adhesin (*ace*) are virulence proteins carried by *E. faecium* that are linked to host invasion, persistence, biofilm formation, and pathogenicity [9]. Moreover, gelatinase is an extracellularly expressed zinc metalloprotease that hydrolyzes casein, collagen, and gelatin [10]. As a result, enterococcal strains considered for use as probiotics should be thoroughly tested for effectiveness and safety [11].

Emerging multidrug-resistant bacterial infections are a significant problem in developing worldwide public health risks [12]. Vancomycin-resistant *E. faecium* is the second most common pathogen on the World Health Organization's (WHO) priority list of antibiotic-resistant pathogens that seriously threaten public health [13]. The expression of *van* operons confers vancomycin resistance. Eight acquired vancomycin-resistance operons (*vanA, vanB, vanD, vanE, vanG, vanL, vanM, *and *vanN*) were described and named according to their ligase genes. Besides, the "accessory" genes *vanY* and *vanZ* are carried by the transposon *Tn1546* and are located on the gene cluster *vanA*. Vancomycin resistance is expressed through three primary resistance genes: *vanH*, *vanA*, and *vanX* (*vanHAX)*. *VanR* and *vanS* are additional essential regulatory genes that govern the primary resistance genes [14, 15]. The *vanA-*positive and *vanB*-positive *enterococci* are the most common European resistance genotypes (14). *Enterococci* expressing *vanA* exhibited elevated resistance to vancomycin and teicoplanin, while *enterococci* expressing *van*B showed elevated resistance to vancomycin only [14].

The health sector and veterinary practice must regularly conduct antimicrobial sensitivity tests and utilize antibiotics appropriately [16, 17]. Combining classical and molecular diagnostic assays offers a precise epidemiological tool for pathogen research. Worryingly, multidrug-resistant bacteria are portrayed as a public health issue [18]. Therefore, this study aimed to investigate the potential risk of companion healthy dogs in the transmission of *enterococci*. Accordingly, antimicrobial-resistant genes, bacteriocin, and virulence genes of *enterococci* collected in Egypt were identified, besides analyzing the phylogenetic relationships among *vanB* and *tetL* genes expressed by *E. faecium* isolated from different species, including humans.

## Results

### Isolates

Ten putative *E. faecium* strains isolated from fecal samples of healthy dogs were characterized by morphology, biochemical tests, and the Api20 Strep system (Biomérieux, France).

### Phenotypic characteristics of the recovered isolates

The Gram stain results revealed a single, pair, or chain of gram-positive cocci, ovoid to coccobacillary in shape. Colonies on blood agar are 1 to 2 mm in diameter, non-hemolytic or alpha-hemolytic; colonies on bile esculin media cause blacking of the medium around the growth. Besides, isolates are catalase-negative and resistant to 6.5% sodium chloride (positive).

**Fig. 1:**
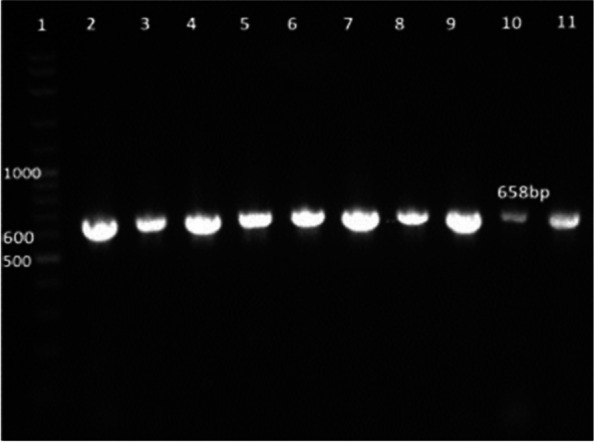
Amplification of a 658 bp gene fragment specific for *E. faecium* by PCR separation by agarose gel electrophoresis. Lane 1, 100 bp ladder; Lanes 2–11, representative *E. faecium* isolates

All isolates were confirmed using a species*-*specific primer for *E. faecium* by the detection of a specific 658-bp PCR product, as shown in Fig. 1.

### Antibiotic sensitivity

The antibiotic sensitivity test showed that 80% of isolates showed phenotypical resistance to vancomycin, and 50% showed resistance to tetracycline, while 30%, 60%, and 10% showed resistance against ceftriaxone, ampicillin, and amoxicillin/clavulanic acid, respectively. All isolates were sensitive to ciprofloxacin.

### Detection of antibiotic resistance, virulence, and enterocin genes using PCR

**Fig. 2:**
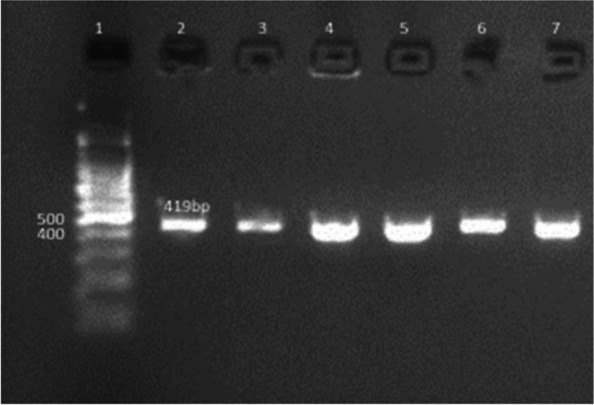
Amplification of a 419-bp fragment of the *gelE* virulence gene by PCR and separation by agarose gel electrophoresis. Lane 1, 100 bp ladder; Lanes 2–7, representative *E. faecium* isolates

**Fig. 3:**
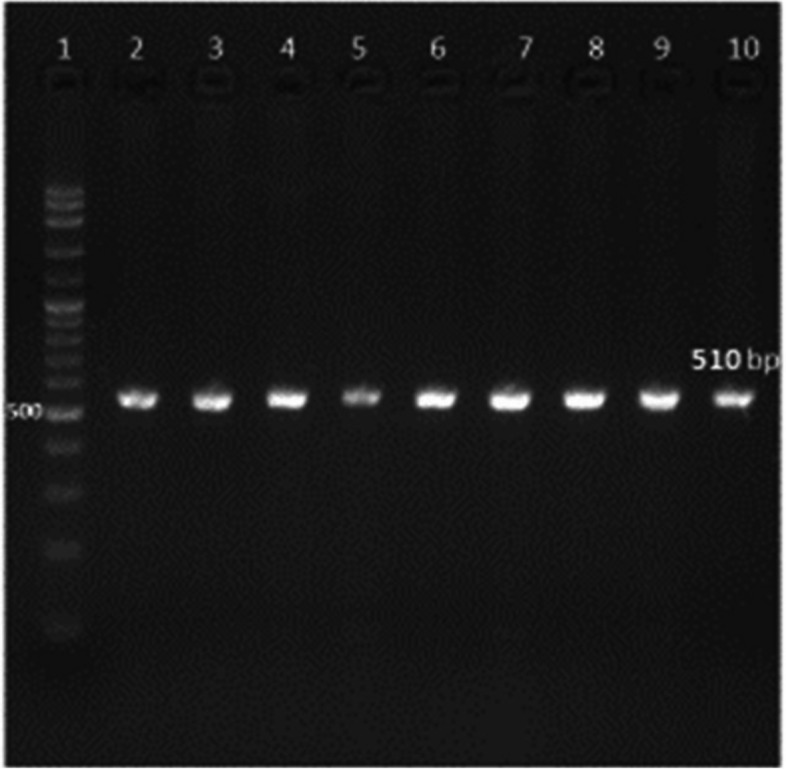
Amplification of a 510-bp fragment of the *esp* virulence gene by PCR and separation by subsequent agarose gel electrophoresis. Lanes 2–10, representative *E. faecium* isolates, Lane 1, 100 bp ladder

**Fig. 4:**
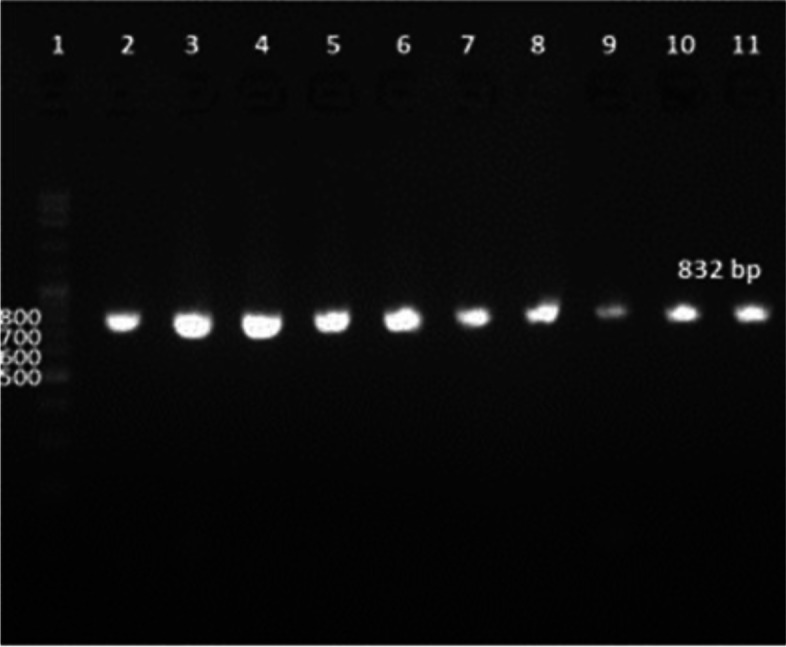
Amplification of an 832 bp fragment of the *vanB* antibiotic resistance gene by PCR and separation by agarose gel electrophoresis. Lanes 2–11, representative *E. faecium* isolates, Lane 1, 100 bp ladder

**Fig. 5:**
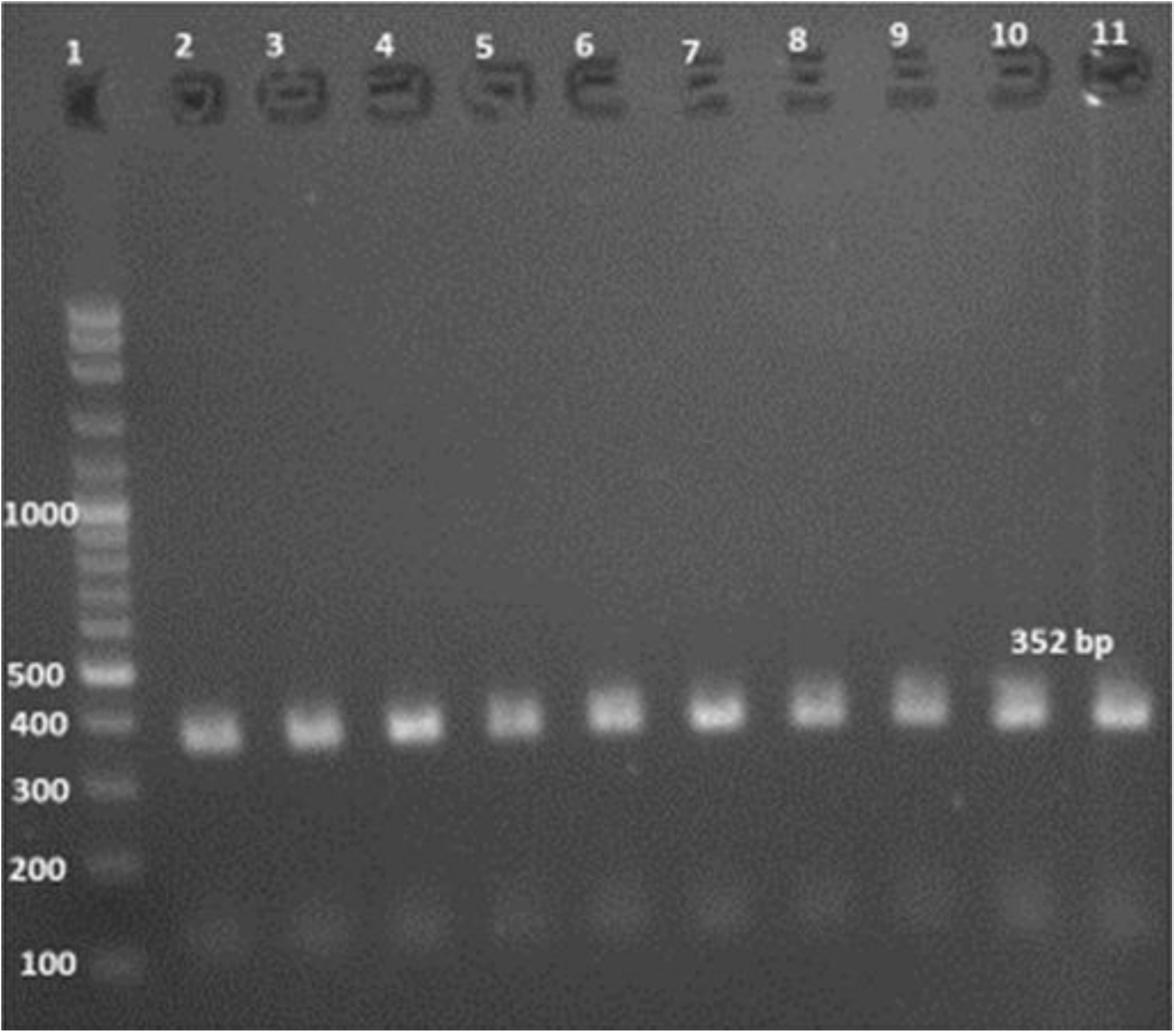
Amplification of a 352 bp fragment of the *tetL* antibiotic resistance gene by PCR and separation by agarose gel electrophoresis. Lane M, 100 bp ladder. Lanes 2-11, representative *E. faecium* isolates

**Fig. 6:**
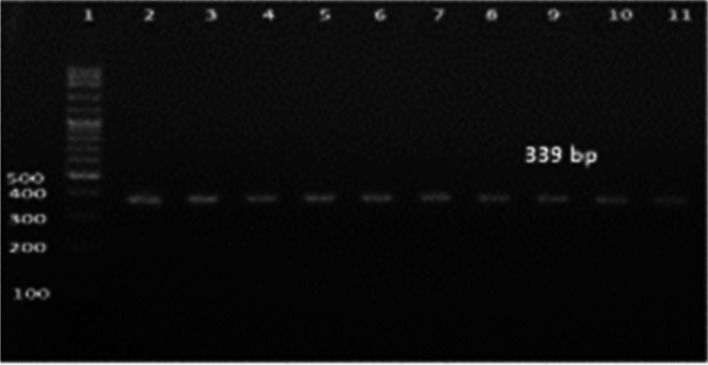
Amplification of a 339 bp fragment of *Enterocin AS-48* by PCR and separation by agarose gel electrophoresis. Lane 1, 100 bp ladder; Lanes 2–11, representative *E. faecium* isolates

**Fig. 7:**
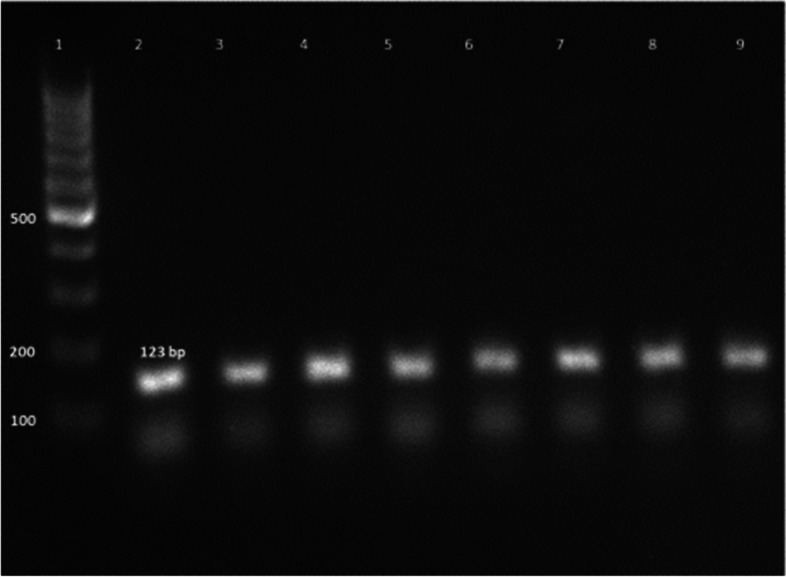
Amplification of a 123 bp fragment of bacteriocin 31 by PCR and separation by agarose gel electrophoresis. Lane 1, 100 bp ladder; Lanes 2–9, representative *E. faecium* isolates

**Fig. 8:**
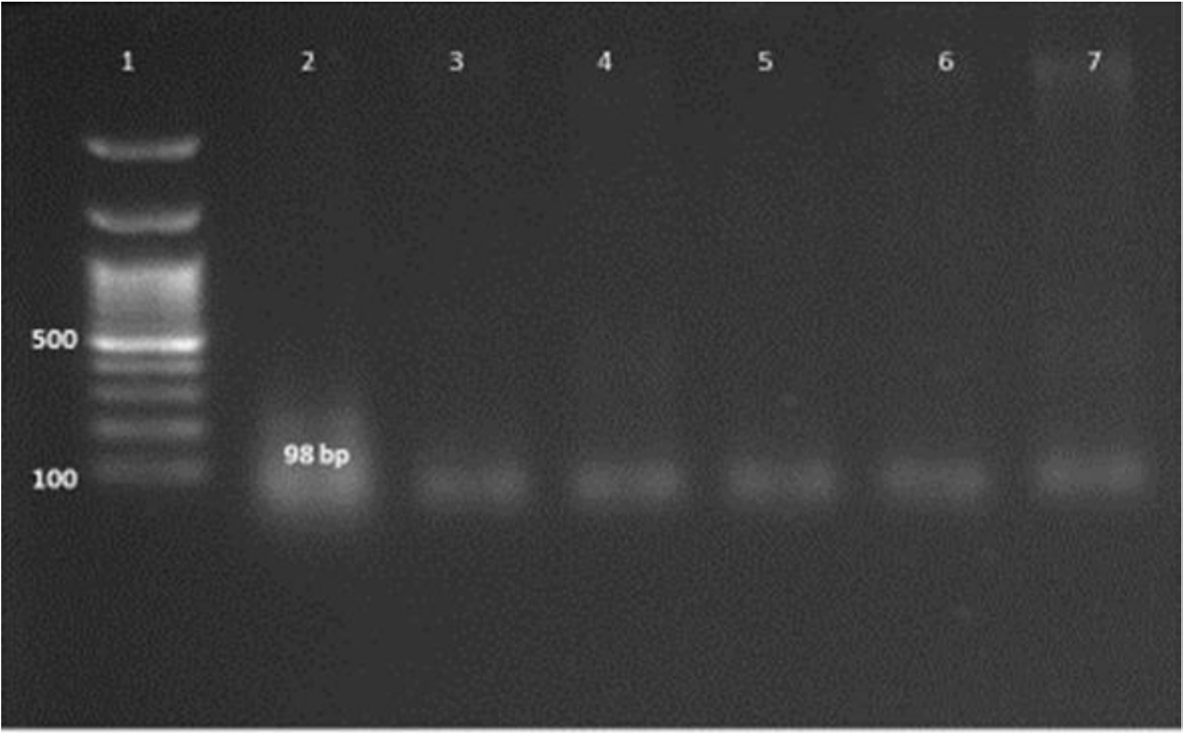
Amplification of a 98 bp fragment of the *Ent L50* gene by PCR and separation by agarose gel electrophoresis. Lane 1, 100 bp ladder; Lanes 2–7, representative *E. faecium* isolates

Ninety percent of *E. faecium* isolates harbored the virulence genes *gelE* and *esp.* In addition, all isolates showed 100% resistance against the antibiotic resistance genes *tetL* and *vanB*. Furthermore, *Ent As-48*, *bacteriocin 31,* and *Ent L50* were found to have a prevalence of 100%, 80%, and 60%, respectively, while none of the isolates expressed *Ent P* or *Ent 1071A/1071B*. These findings are summarized in Table 1 and Figs. 2, 3, 4, 5, 6, 7 and 8.

**Table 1:** Results of PCR targeting virulence, antibiotic resistance, and enterocin genes in *E. faecium* isolates

_*E. faecium* isolates_	_*Phenotypic resistance*_	_*gelE*_	_*esp*_	_*tetL*_	_*vanB*_	_*Ent As-48*_	_*Bacteriocin 31*_	_*Ent L50*_	_Significant_
_1_	_**VAN**_	_**+**_	_**+**_	_**+**_	_**+**_	_**+**_	_**+**_	_**+**_	_**N.S**_
_2_	_**T, CRO**_	_**+**_	_**+**_	_**+**_	_**+**_	_**+**_	_**+**_	_**+**_	_**N.S**_
_3_	_**VAN**_	_**+**_	_**+**_	_**+**_	_**+**_	_**+**_	_**+**_	_**+**_	_**N.S**_
_4_	_**VAN,T, AMP**_	_**+**_	_**+**_	_**+**_	_**+**_	_**+**_	_**+**_	_**_**_	_**_
_5_	_**VAN, T, AMP**_	_**_**_	_**+**_	_**+**_	_**+**_	_**+**_	_**_**_	_**+**_	_**_
_6_	_**VAN, CRO**_	_**+**_	_**+**_	_**+**_	_**+**_	_**+**_	_**+**_	_**+**_	_**N.S**_
_7_	_**VAN, T, AMP**_	_**+**_	_**+**_	_**+**_	_**+**_	_**+**_	_**+**_	_**+**_	_**N.S**_
_8_	_**VAN, AMP**_	_**+**_	_**+**_	_**+**_	_**+**_	_**+**_	_**+**_	_**_**_	_**_
_9_	_**VAN, AMP**_	_**+**_	_**+**_	_**+**_	_**+**_	_**+**_	_**+**_	_**_**_	_**_
_10_	_**VAN, T, AMC, CRO, AMP**_	_**+**_	_**_**_	_**+**_	_**+**_	_**+**_	_**_**_	_**_**_	_**_
_Percentage_		_**(9/10) 90%**_	_**(9/10) 90%**_	_**10/10 100%**_	_**(10/10)**_	_**(10/10) 100%**_	_**(8/10) 80%**_	_**(6/10) 60%**_	
_Significant_		_**_	_**_	_**N.S**_	_**N.S**_	_**N.S**_	_**_	_**_	

*VAN* Vancomycin, *CIP* Ciprofloxacin, *T* Tetracycline, *AMC* Amoxicillin /Clavulanic acid, *CRO* Ceftriaxone, *AMP* Ampicillin

^**^ Significant at *P* ≤ 0.01 N.S. non-significant

### Phylogenetic analysis

**Fig. 9:**
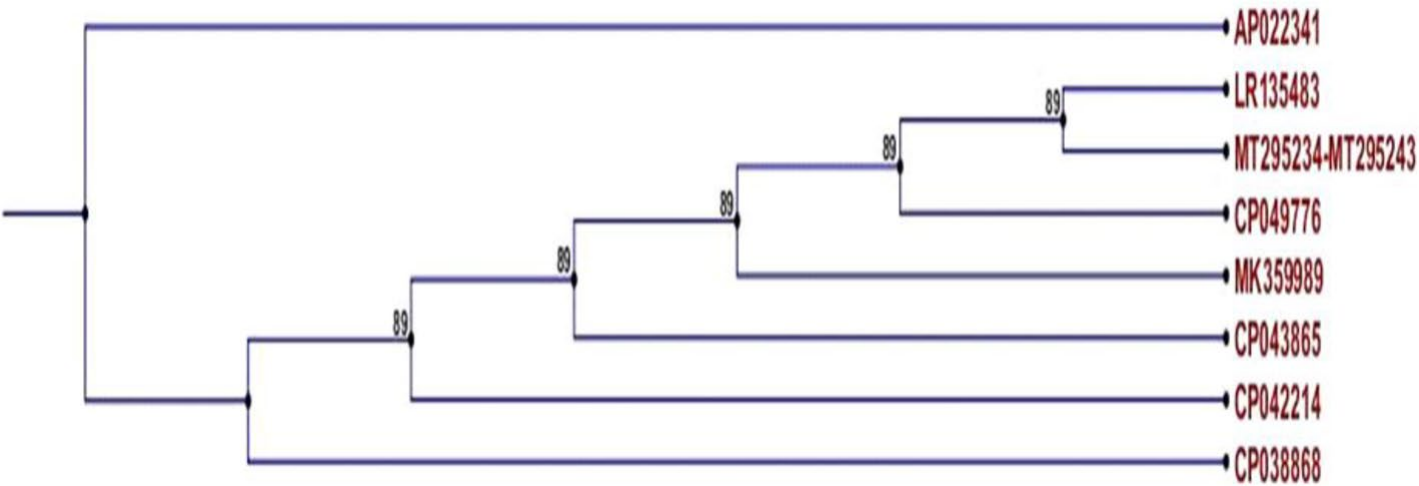
Phylogenetic tree of representative *tetL* nucleotide (sequences (GeneBank accession numbers MT295234—MT295243) from *E. faecium* isolates and reference sequences, constructed using the neighbor-joining method

The *tetL* sequences of all isolates showed high homology with reference sequences from *E. faecium* (LR145483), *E. faecalis* (CP049776), and *Strept. suis* (MK359989) (Fig. 9).

**Fig. 10:**
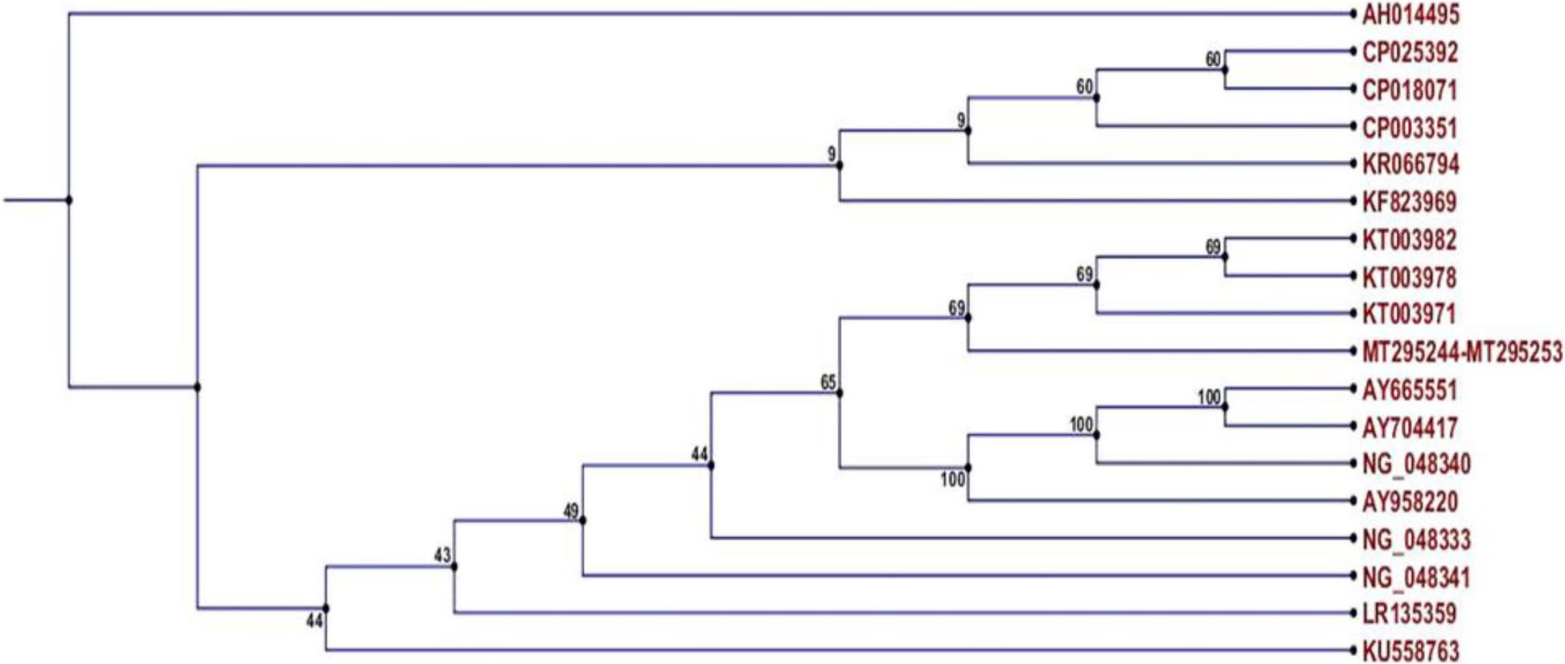
Phylogenetic tree of *vanB* sequences from dog *E. faecium* isolates *(GenBank accession numbers* MT295244—MT295253) and reference sequences, constructed using the neighbor-joining method

Moreover, all *vanB* sequences from dog isolates formed a distinct clade with *VanB* sequences of *E. faecium* isolates from humans (KT003971, KT003978, and KT003982) (Fig. 10).

## Discussion

Enterococcus opportunistic pathogens are a significant public health concern due to their frequent involvement in nosocomial infections, high antibiotic resistance, and severe morbidity. This study suggests that *enterococci* carrying antibiotic resistance and virulence genes can spread through asymptomatic pets. Our results reflect that VRE may be spread in households and veterinary hospitals through the feces of healthy dogs. Five *E. faecalis* and 15 *E. faecium* isolates were recovered from 20 *Enterococcus* isolates taken from the library collections of randomly chosen private hospitals in El Qanater El Khayreya, Egypt, suggesting that these strains are common contaminants in some Egyptian hospitals [19]. In addition, 61 *E. faecium* strains were identified among 110 isolates from clinical specimens [20]. Isolates of *E. faecium* have previously been found in healthy dogs, although at a lower frequency than in the current study. Said and his colleagues [21] identified 16 *E. faecium* strains among 39 isolates from healthy dogs. Moreover, Feßler et al*.* [22] found 37 *E. faecium* strains among 215 samples collected from dogs and cats. These findings indicate that *Enterococcus* species distribution varies depending on the host environment.

Various factors, including expression of *esp* and *gelE,* control the virulence of *enterococci.* However, the expression of these virulence genes varies widely among enterococcal species from different hosts. The *esp* is responsible for increased biofilm formation and colonization potential. At the same time, *gelE* is essential for resistance against the host's innate immune defense [4]. The *esp* was detected in 66.4% and the *gelE* in 33.6% of clinical specimens [20].

On the other hand, *gelE* and *esp* were not found in any of the eight *E. faecium* isolates from healthy dogs in Egypt [23]. The *gelE* was found in 35.41% of isolates from wild and domestic ruminants in Italy, the highest frequency among tested genes, while *esp* the least frequent, found in only 0.04% of samples [24]. Besides, *gelE* was found in the vast majority of samples (91.4%), consistent with our findings, but *esp* was found less frequently (65.7%) than in the present study (90%) [25]. Similarly, a study conducted at the University Hospital of the Faculty of Veterinary Medicine, Cairo University, and private veterinary clinics in Cairo [26] reported oral colonization of domestic dogs and cats by *E. faecalis* and *E. faecium*. However, only two of nine canine ampicillin-resistant *E. faecium (*AREfm) multidrug-resistant isolates and none of the feline AREfm isolates expressed the *esp* gene. These findings suggest that virulence genes vary substantially across *E. faecium* strains. Therefore, screening is recommended for all strains isolated in clinical settings (Table 1).

Regarding phenotypic examination for antibiotic resistance, the results showed that not all the isolates with resistance genes showed phenotypic resistance. These findings confirmed that isolates may carry antibiotic resistance genes without expression (Table 2).

**Table 2:** The occurrence of MDR (Multidrug resistance) among the recovered isolates

No. of Isolates	%	Type of Resistance	Phenotypic Resistance	Resistance Genes
2	20%	R	VAN	*VanB* *tetL*
1	10%	R	TE CRO	*VanB* *tetL*
1	10%	R	CRO	*VanB* *tetL*
2	20%	R	VAN AMP	*VanB* *tetL*
3	30%	MDR	VAN TE AMP	*VanB* *tetL*
1	10%	MDR	VAN TE AMC	*VanB* *tetL*

Furthermore, vancomycin-resistant *enterococci* have been identified as human pathogens in the natural environment, and the spread of opportunistic bacteria with vancomycin resistance outside of the hospital environment poses a severe public health risk because vancomycin is considered the last line of defense. Ulrich and his colleagues [27] have documented 35 VRE outbreaks, with 757 individual infections and 77 deaths. The most common recovery sites were rectal swabs and fecal samples, suggesting that these infections spread due to poor hygiene. Further, previous antibiotic treatment was the most significant risk factor.

Concerning the *van*B gene, our result is consistent with studies showing that *E. faecium* can colonize the gut, although typically without substantial vancomycin resistance (MIC = 3 mg/L). Antibiotic resistance is far more frequent in this study than previously reported. According to El-Tayeb et al. [28], only 24% of 25 VRE isolates collected from diverse regions in Egypt were *E. faecium.* Moreover, Seputiene et al. [29] identified the *tetL* gene in only 23% of isolates from *E. faecium-*infected farm animals (pigs, cattle, and poultry).

Similarly, *tetL* was found in only 12% of isolates collected from 2 hospitals in Kerman, Iran [30]. In Japan, *tetL* was found in 30.4% of *E. faecium* isolates obtained from clinical samples [31]. The high proportion of *vanB*- and *tetL*-expressing strains isolated in this study is suggested to be due to the inappropriate use of antibiotics in veterinary medicine. For instance, *tetL* was identified in 25 out of 31 *Enterococcus* isolates collected from infected poultry in six regions of Egypt [32]. In turn, these animals could potentially spread the contamination into the surrounding environment, resulting in both animal and human infections. Most of the strains isolated in this study harbored a combination of *Ent As-48, bacteriocin 31, *and *EntL50 *genes, while neither *Enterocin* 1071A/1071B nor *Enterocin* P was found in any isolate.

Similarly, four strains of eight *E. faecium* from eight healthy dogs in Egypt carried the *Ent AS-48* gene, and one strain carried the *EntL50A/B* gene [23].

In contrast, *enterocin* AS-48 was not found among 54 strains from different origins, including animals, while combinations including *EntP* and *Ent*L50A*/B* were the most common (44%) [33]. Generated peptides with masses similar to those of enterocins A and B from 3 *E. faecium* isolates were found in donkey milk [34]. According to the previous study, wild animals are a significant source of bacteriocinogenic *enterococci*, especially in fecal matter, with *enterocin P *being the most common in most isolates; however, only a subset of bacteriocin genes is expressed [35].

Sequence alignment of *tetL* genes from our isolates with various reference sequences, including human isolates, revealed significant homology with *E. faecium* (LR145483), *E. faecalis* (CP049776), and *Strept. suis* (MK359989). Moreover, the *vanB* expressed by these isolates formed a separate clade with *vanB* sequences expressed by *E. faecium* isolated from humans (KT003971, KT003978, and KT003982), which suggests that *tetL* and *vanB* resistance genes can be transferred between dogs and their owners.

## Conclusion

Companion animals are a potential source of severe VRE infections that can endanger veterinary health and human health. The findings reflected that *E. faecium* isolates from domestic dogs in Egypt frequently harbor antibiotic resistance genes and virulence factors. Thus, an effective antimicrobial stewardship program and regular surveillance using a transdisciplinary "One Health" approach are recommended to investigate the role of dogs as vectors for vancomycin resistance and prevent its dissemination.

## Materials and methods

### Sample collection

Fresh fecal samples were obtained from 10 randomly selected healthy domestic dogs admitted to the Faculty of Veterinary Medicine (Cairo University, Egypt), for routine medical checkups or vaccinations from January to November 2021. Sampled dogs were of both sexes. Samples were taken using sterile swabs and delivered in an icebox to the National Research Centre Microbiology and Immunology Laboratory for immediate processing.

### Bacterial isolation and identification

Fecal swabs were pre-diluted, added to a 25 mL Ringer's solution containing 0.30 g/L of potassium chloride, 0.33 g/L of calcium chloride dehydrate, and 8.60 g/L of sodium chloride, and shaken vigorously for 30 min. Ten milliliters of the resultant suspension were added to 90 ml of nutrient broth (Merck). Incubation of the inoculated media was performed for 48 h at 37 °C. The inoculum was next streaked over bile esculin agar (Oxoid, Hampshire, UK) and incubated under the same conditions [36]. An analysis of colony morphology on blood agar prepared from tryptic soy agar (Oxoid, Hampshire, UK) with 5% sheep blood was performed. Moreover, catalase expression and resistance to 6.5% sodium chloride were examined*.* Biochemical characteristics were analyzed using the Api20 Strep system (Biomérieux, France).

### Antibiotic susceptibility test

Kirby Bauer's disc diffusion method [37] was used to investigate the antibiotic sensitivity of *Enterococcus faecium* isolates against different antimicrobial categories such as glycopeptides (Vancomycin 30 µg), tetracycline (Tetracycline 30 µg), fluoroquinolones (Ciprofloxacin 5 µg), cephalosporin (Ceftriaxone 30 µg), beta-lactamase inhibitors (Amoxicillin/Clavulanic acid 30 µg), and penicillins (Ampicillin 10 µg). All antibiotic discs were obtained from HI Media Laboratories (Mumbai, India). 20 µL overnight culture (1 × 10^5^ CFU/ml) was added to 100 mL of Nutritional Broth (NB; Oxoid, UK) medium and incubated for 24 h at 37°C/120 rpm. Using cotton swabs, a bacterial culture (100 µL) was streaked onto Mueller Hinton Agar (MHA; Oxoid, UK) plates and incubated aerobically at 37°C for 18–24 h. The inhibitory zones (mm) were measured after the incubation, and the findings were classified according to the CLSI interpretation criteria [37].

### Detection of virulence, antibiotic resistance, and bacteriocin genes using PCR

The QIAamp DNA Mini kit (Qiagen, Germany, GmbH) was used to extract genomic DNA from samples according to the manufacturer's instructions, and the nucleic acid was eluted with 50 µl of elution buffer.

All PCR reactions were conducted in a final reaction volume of 25 μL containing 12.5 μL 2 × cosmo PCR red master mixes (Cat. W1020300X, Willofort Co., UK), 1 μL (10 μM) of each primer (Metabion, Germany), and 1 μL of sample DNA. The PCR products were separated by electrophoresis on 1.5% agarose gels, which were then photographed and analyzed using the InGenius3 gel documentation system (Syngene, UK). The primer sequences and annealing temperatures used for PCR are presented in Table 3.

**Table 3:** Target genes, primer sequences, amplicon sizes and cycling conditions for PCR

Target gene	Primers sequences	PCR product	Anneal. Temp	Reference
*E. faecium (16 s rRNA)*	F: TTGAGGCAGACCAGATTGACG R: TATGACAGCGACTCCGATTCC	658 bp	54 °C	[38]
Enterococcal surface protein (*esp*)	F: TTGCTAATGCTAGTCCACGACC R: GCGTCAACACTTGCATTGCCGAA	510 bp	54 °C	[39]
Gelatinase (*gel*E)	F: ACCCCGTATCATTGGTTT R: ACGCATTGCTTTTCCATC	419 bp	54 °C	[40]
Enterocin AS-48	F: AGAGTTTGATCMTGGCTCAG R: ACGGYTACCTTGTTACGACTT	339 bp	53 °C	[41]
Bacteriocin 31	F: TAT TAC GGA AAT GGTTTATATTGT R: TCTAGG AGC CCA AGG GCC	123 bp	53 °C	[41]
Enterocin L50 A/B	F: TGG GAG CAATCG CAA AAT TAG R: ATT GCC CAT CCT TCT CCA AT	98 bp	53 °C	[42]
Enterocin P	F: TAT GGT AAT GGT GTT TAT TGTAAT R: ATG TCC CATACC TGC CAAAC	120 bp	53 °C	[41]
Enterocin 1071A/1071B	F: CCTATT GGG GGA GAG TCG GT R: ATA CAT TCT TCC ACT TAT TTT T	343 bp	53 °C	[43]
*tet*L	F: AGCTGCATTTCCAGCACTCG R: CAGGAATGACAGCACGCTAAC	352 bp	55 °C	[44]
*Van*B	F: CATGATGTGTCGGTAAAATC R: ACCGGGCAGRGTATTGAC	832 bp	55 °C	[44]

The genes of the *E. faecium* confirmation, entertains, and antimicrobial resistance indicated in Table 1 were amplified using the following thermocycling: 94°C for 5 min, 35 cycles of 94°C for 1 min, 53°C, 54°C, and 55°C (according to specific annealing for each gene) for 1 min, and 72°C for 40 s, and a final extension step at 72°C for 7 min.

### Phylogenetic tree construction

Ten *tetL* nucleotide sequences from *E. faecium* isolates were submitted to GenBank under accession numbers MT295234 to MT295243 (https://www.ncbi.nlm.nih.gov/nuccore/MT295234), and ten *VanB* nucleotide sequences from *E. faecium* isolates were submitted to GenBank under accession numbers MT295244 to MT295253 (https://www.ncbi.nlm.nih.gov/nuccore/MT295244).

Both *vanB* and *tetL* genes from isolates were sequenced using 3730 L sequencers (Applied Biosystem, USA) at Macrogen (Seoul, Korea), and findings were validated by two-directional sequencing using the same forward and reverse PCR primers listed in Table 3. The gene sequences were analyzed using BioEdit 7.0.4.1 and ClustalW2 (http://www.clustal.org/), and compared to reference sequences of *Enterococcus* spp. using a neighbor-joining application in CLC Sequence Viewer 6.

### Statistical analysis

Data were computerized and analyzed by the SPSS program (2004) [45]. Moreover, significant differences among means were detected by Duncan (1955) [46]:

$${\mathrm{Y}}_{\mathrm{ijk}} =\upmu + {\mathrm{E}}_{\mathrm{i}} + {\mathrm{P}}_{\mathrm{j}} + + {\left(\mathrm{E}\times \mathrm{P}\right)}_{\mathrm{ij}} + {\mathrm{e}}_{\mathrm{ijk}}$$

where Y_ijk_: Observation of i *E. faecium* isolates, and j phenotypic resistance; µ: General mean; E_i_: Fixed effect of *E. faecium* isolates; P_j_: Fixed effect of (D_j_) phenotypic resistance; (S × D)_ij_: Effect of interaction (S × D)_ij_; and e_ijk_: Residual effect.

## Data Availability

Ten *tetL* nucleotide sequences from *E. faecium* isolates were submitted to GenBank under accession numbers MT295234 to MT295243 and are publicly available at https://www.ncbi.nlm.nih.gov/nuccore/MT295234. Furthermore, ten *VanB* nucleotide sequences from *E. faecium* isolates were submitted to GenBank under accession numbers MT295244 to MT295253 and are publicly available at https://www.ncbi.nlm.nih.gov/nuccore/MT295244.
